# Hemostatic Status of Neonates with Perinatal Hypoxia, Studied via NATEM in Cord Blood Samples

**DOI:** 10.3390/children11070799

**Published:** 2024-06-29

**Authors:** Marina Tsaousi, Rozeta Sokou, Abraham Pouliakis, Marianna Politou, Nicoletta Iacovidou, Theodora Boutsikou, Alma Sulaj, Eleni Karapati, Andreas G. Tsantes, Argirios E. Tsantes, Serena Valsami, Zoi Iliodromiti

**Affiliations:** 1 Neonatal Department, Aretaieio Hospital, School of Medicine, National and Kapodistrian University of Athens, 11528 Athens, Greece; martsaousi@med.uoa.gr (M.T.); rosesok@med.uoa.gr (R.S.); theobtsk@med.uoa.gr (T.B.); alsulaj@med.uoa.gr (A.S.); helenakar@med.uoa.gr (E.K.); ziliodromiti@med.uoa.gr (Z.I.); 2 2nd Department of Pathology, “Attikon” University Hospital, School of Medicine, National and Kapodistrian University of Athens, 12462 Athens, Greece; apouliak@med.uoa.gr; 3 Hematology Laboratory Blood Bank, Aretaieio Hospital, School of Medicine, National and Kapodistrian University of Athens, 11528 Athens, Greece; mpolitou@med.uoa.gr (M.P.); svalsami@med.uoa.gr (S.V.); 4 Laboratory of Haematology and Blood Bank Unit, “Attikon” University Hospital, School of Medicine, National and Kapodistrian University of Athens, 12462 Athens, Greece; agtsantes@med.uoa.gr (A.G.T.); atsantes@med.uoa.gr (A.E.T.)

**Keywords:** thromboelastometry, NATEM, neonates, perinatal hypoxia, cord blood

## Abstract

Background: Perinatal hypoxia may result in coagulation dysfunction. Diminished blood flow or oxygen to the fetus/neonate during the perinatal period can cause bone marrow and liver function impairment, leading to thrombocytopenia, impaired synthesis of clotting and fibrinolytic factors, and increased destruction of platelets in the small blood vessels. The goal of the present study was to evaluate the hemostatic status of newborns with perinatal hypoxia via the non-activated thromboelastometry (NATEM) assay in cord blood samples. Methods: 134 hypoxic neonates born in our maternity unit over a 1.5-year period were enrolled in this observational cohort study, and 189 healthy neonates served as the control group. Participation in the study was voluntary and parents signed informed consent prior to recruitment. Demographic and clinical data were recorded on admission, and the NATEM method was performed on cord blood samples. The following NATEM values were evaluated: clotting time (CT), alpha angle (α-angle), clot formation time (CFT), clot amplitude at 5 and 10 min. (A5, A10), maximum clot firmness (MCF), clot lysis index at 60 min. after CT (LI60), and maximum clot elasticity (MCE). Statistical analysis was conducted utilizing the SAS for Windows 9.4 software platform. Results: Neonates with perinatal hypoxia exhibited decreased fibrinolytic potential in comparison to healthy neonates, as indicated by increased LI60, and this difference was statistically significant (LΙ60: 94 (92–96) Vs 93 (91–95), *p* value = 0.0001). There were no statistically significant differences noted among the remaining NATEM variables. Conclusion: Our findings indicate decreased fibrinolytic potential in hypoxic neonates in comparison to healthy neonates, suggesting that NATEM could serve as an effective tool for promptly identifying hemostasis dysfunction in this group of neonates.

## 1. Introduction

Perinatal asphyxia, also known as birth asphyxia, is a state of a markedly impaired blood gas exchange and/or impaired blood flow to or from the fetus during the perinatal period. If the duration or the intensity of this condition is substantial, it triggers fundamental biochemical alterations, i.e., hypoxia, hypercapnia, and consequent acidosis. The pathophysiology of perinatal asphyxia is quite complex; it may be due to an insult occurring prior to, during, or after delivery and may be secondary to obstetrics or fetal/neonatal risk factors [1]. The American College of Obstetricians and Gynecologists (ACOG) task force on neonatal encephalopathy, along with the American Academy of Pediatrics (AAP), published a report delineating neonatal signs and contributing factors, used to define an acute hypoxia-ischemia event occurring in the peripartum or intrapartum period that may contribute to the manifestation of neonatal encephalopathy [2,3].

A similar term, which is sometimes (inaccurately) used interchangeably with the term “perinatal asphyxia”, is “non-reassuring fetal status” (NRFS), previously known as “fetal distress”. NRFS describes a transient or persistent interruption of oxygen supply to the fetus, which leads to progressive fetal hypoxia and presumably acidosis, yet does not axiomatically result in birth asphyxia, according to the criteria defined by the AAP and ACOG [4].

If the hypoxia is of sufficient length or severity, the fetal homeostatic compensatory mechanisms may be overwhelmed and several complications may arise. Hypoxic-ischemic encephalopathy, which leads to major neurodevelopmental sequelae, constitutes the most severe consequence of perinatal asphyxia [1,5]. Other than brain damage, multiple extracerebral organs may be involved; among them, the hemostatic system is commonly affected. Perinatal hypoxia is considered one of the major causes of thrombocytopenia during the perinatal period, and according to the literature, several mechanisms are involved. Reduced oxygenation of the bone marrow appears to decrease platelet production primarily by inhibiting megakaryocyte synthesis and function [6,7,8,9]. Disseminated intravascular coagulation (DIC), predominantly initiated by the release of tissue factor from the ischemic damage to the endothelium, may lead to the destruction of platelets in the small blood vessels, as well as to exaggerated consumption of clotting factors, therefore increasing bleeding diathesis [10,11,12,13]. Additionally, systemic oxygen deprivation may impact liver function, the major site of synthesis of blood coagulation and fibrinolytic factors, therefore disturbing the hemostatic balance of the hypoxic neonates [14,15,16]. Consequently, numerous cases of thrombotic or hemorrhagic complications have been reported in the literature [17,18,19].

Prompt diagnosis and management, or preferably prevention of bleeding or thrombotic events, is necessary to reduce morbidity and mortality rates of hypoxic neonates. Monitoring of the hemostatic status typically commences with conventional coagulation tests, such as PT, aPTT, and INR. Although these test results are frequently found to be abnormal in hypoxic neonates, their ability to diagnose hemorrhagic complications is frequently questioned, since they provide only a vague assessment of the coagulation cascade without taking into account the interaction of pro- and anti-coagulant factors with cellular elements of hemostasis. Conversely, viscoelastic whole blood analyses, including thromboelastography (TEG) and (rotational) thromboelastometry (ROTEM, TEM), seem an appealing alternative to conventional coagulation tests, since they provide a point-of-care analysis of clot formation, stabilization, and lysis, needing only a small amount of blood. Initial results can be obtained within 10 min., facilitating prompt diagnosis and enabling timely therapeutic decisions [20,21,22]. The implementation of TEG/ ROTEM in neonates remains limited since reference values have not been established yet. A few studies can be found in the literature regarding the use of TEG/ ROTEM in healthy newborns [23,24,25,26,27,28,29,30,31,32] and in neonates with an underlying medical condition, mostly those with sepsis, intracranial hemorrhage, cardiac surgery, and IUGR neonates [33,34,35,36,37,38,39,40,41,42,43,44]. Most of these studies performing ROTEM were conducted using extrinsically-activated thromboelastometry (EXTEM) or intrinsically-activated thromboelastometry (INTEM). However, emerging evidence suggests that the non-activated thromboelastometry (NATEM) method may be more sensitive in reflecting the in vivo hemostatic cascade. Unlike INTEM and EXTEM, which use ellagic acid and tissue factor, respectively, to activate the coagulation cascade, the NATEM method is initiated solely by the addition of calcium. Consequently, it exhibits high sensitivity to any endogenous factor activating hemostasis, such as the circulating tissue factor in cases of hypoxia, DIC, sepsis, or trauma [22,45,46,47]. Additionally, the existing literature demonstrates that NATEM assay is also highly sensitive in identifying fibrinolysis derangement [48]. Studies with NATEM have been primarily conducted on adult populations [49]. Regarding the neonatal population, a limited number of studies were identified, involving both healthy [23,50] and ill newborns [43,51,52]. As for neonates with perinatal hypoxia, studies using either TEG or EXTEM have been found; no studies focusing on the use of NATEM in hypoxic newborns have been retrieved [10,53,54,55,56].

We aimed to investigate the coagulation profile of neonates with perinatal hypoxia via NATEM assay in cord blood samples.

## 2. Materials and Methods

### 2.1. Study Design and Participants

**Figure 1:**
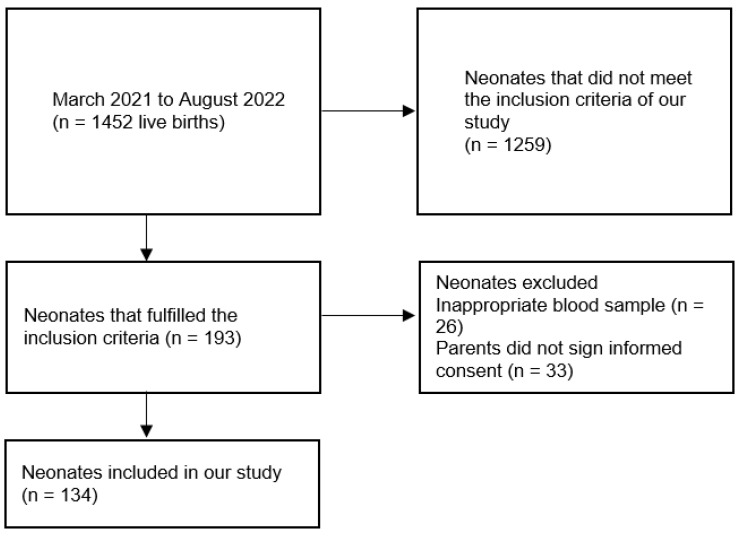
Research group flowchart.

This prospective observational study was carried out in the Neonatology Department of Aretaieio Hospital, Athens, Greece. Neonates with perinatal asphyxia or NRFS born in our maternity unit over a 1.5-year period (between March 2021 and August 2022) comprised the study population. Data describing the recruitment process for our study are displayed in the flowchart (Figure 1). One hundred and eighty-nine (189) healthy neonates, previously recruited by our research group to define reference values for the NATEM method in umbilical cord blood samples, served as controls [50]. Inclusion criteria for newborns with perinatal hypoxia were the following:

I. Neonates with perinatal asphyxia, as defined by the ACOG and AAP guidelines. According to these guidelines, neonatal signs consistent with a critical hypoxic-ischemic intrapartum or peripartum event are the following: (i) persistence of low Apgar scores at 5 and 10 min. (Apgar score < 5 at 5 and 10 min., respectively), (ii) profound fetal acidemia (umbilical artery pH < 7 or BD > −12 mmol/L or both), (iii) neuroimaging findings on brain MRI (magnetic resonance imaging) or MRS (magnetic resonance spectroscopy) compatible with acute hypoxia-ischemia, and iv) presence of multiple organ dysfunction. Potential contributing factors mentioned in the report are the following: (i) a sentinel hypoxic or ischemic event occurring with close proximity to labor or delivery, (ii) fetal heart rate tracing compatible with an acute event, (iii) brain injury findings on diagnostic imaging studies characteristic of an acute intrapartum or peripartum event, and (iv) lack of other significant risk factors that could contribute to the pathogenesis of neonatal encephalopathy [2].

II. Neonates with NRFS meeting at least 2 of the following antepartum and/or at least 3 of the following postpartum criteria.

Antepartum criteria: (i) placental abruption or placental insufficiency, uterine rupture, umbilical cord compression, or reduced amniotic fluid, (ii) non-reassuring or abnormal non-stress test (NST), including abnormal fetal heart rate (>180 bpm or <110/min) or fetal arrhythmia, that persist despite medical interventions (e.g., maternal intravenous hydration, oxygen administration, or positioning mother in the left lateral position), (iii) decreased fetal movements, (iv) meconium-stained liquor.

Postpartum criteria: (i) cord blood pH between 7 and 7.25, (ii) base excess < −7 mmol/L, (iii) lactic acid > 4 mmol/L, (iv) clinical signs observed within the first 72 h of life, including subtle and transient signs of respiratory distress, poor feeding, bloody gastric content, and high-pitched cry, (v) laboratory findings, such as moderately increased SGOT (serum glutamic-oxaloacetic transaminase), CK (creatine kinase), LDH (lactate dehydrogenase), troponin-I, or CRP (C-reactive protein) without clinical signs compatible with infection or the presence of NRBCs (nucleated red blood cells) in the peripheral smear.

Exclusion criteria were genetic syndromes or congenital malformations, perinatal infection, family history of coagulation disorders, and unsuitable blood samples (inadequate sample quantity or detection of clot within the sample).

Data regarding demographics, maternal history and medication intake, pregnancy complications, as well as neonatal complications (e.g., neonatal resuscitation, transfer to neonatal intensive care unit (NICU), respiratory distress syndrome (RDS), thrombotic or hemorrhagic complications) were recorded in an electronic file. All newborns received intramuscularly 1 mg of vitamin K and were clinically assessed until discharge. Specifically, vital signs and clinical examination were performed twice daily and when considered necessary (i.e., signs of infection, jaundice, feeding difficulties, respiratory distress) laboratory tests were obtained. Additionally, brain, abdominal, and renal ultrasounds were performed on all hypoxic neonates before discharge from the hospital.

Based on the minimal handling policy of our maternity unit, neonatal blood samples for laboratory investigations compatible with perinatal stress were exclusively obtained when deemed necessary, according to their clinical course.

### 2.2. Sample Collection and NATEM Assay

Immediately following birth, the umbilical cord was clamped and two blood specimens were drawn from the umbilical vein. One specimen was drawn with a 21-gauge needle in a 1 mL heparinized insulin syringe and was used for umbilical cord pH (GEM Premier 3000, Instrumentation Laboratory, Bedford, MA, US). Another sample was then drawn from the umbilical vein with a 21G needle in a 5 mL syringe and was immediately transferred in a blue-top citrate tube via a Vacutainer system (VACUETTE TUBE 3 mL 9NC Coagulation sodium citrate 3.2%). The tube was then smoothly inverted 5 times to achieve a uniform distribution of the anticoagulant within the blood specimen and was thoroughly checked for the presence of blood clots; if a blood clot was detected, the sample was discarded. The test was performed within 30 min. of sample withdrawal. Three hundred (300) μL of whole blood were tested on the ROTEM^®^ delta analyzer (Tem Innovations GmbH, Munich, Germany) via the NATEM assay using the relevant automated pipette programs in accordance with the manufacturer’s instructions. Blood clotting initiation was triggered by the addition of 20 μL of a 0.2 M calcium chloride solution (star-TEM^®^ 20 reagent) into the cuvette, and the test continued for at least one hour after clot lysis at 30 min. We documented the following NATEM parameters: clotting time (CT, seconds), duration from initiation of the test until a clot firmness amplitude of 2 mm is achieved, clot formation time (CFT, seconds), time between 2 mm and 20 mm clot firmness amplitude, alpha angle (α-angle, α^ο^), angle formed between the baseline and a tangent drawn to the clotting curve at the 2 mm point, reflecting the clot kinetics, clot amplitude at 5 and 10 min. (A5 and A10, mm), maximum clot firmness (MCF, mm), maximal amplitude of clot firmness achieved throughout the test, clot lysis index at 60 min. after CT (LI60, %), proportion of MCF still present at 60 min. after CT, expressed as % of MCF, and maximum clot elasticity (MCE = 100 × MCF/(100 − MCF)).

### 2.3. Statistical Analysis

The SAS for Windows 9.4 software platform (SAS Institute Inc., Cary, NC, USA) (DiMaggio, 2013; SAS Institute, 2014) was used for the statistical processing of the collected data. Descriptive values were reported using the median and Quartile 1 (Q1) to Quartile 3 (Q3) range. Group comparisons for qualitative parameters were based on the chi-square test (and, when necessary, Fisher’s exact test was applied). The normality distribution of the arithmetic data was checked via the Shapiro–Wilk test, and thus, non-parametric tests were performed. In particular, the Mann–Whitney U test was used. Investigation of potential confounding factors was based on multiple linear regression; NATEM parameters were the dependent variables, while asphyxia, birth weight, gestational age, gender, and delivery mode were the independent variables. The level of significance (*p*-value) was set to 0.05, and two-sided tests were performed.

### 2.4. Ethical Approval

Our study protocol was in line with all national policies and was approved by the Institutional Review Board of Aretaieio Hospital, Athens, Greece. Participation in the study was voluntary and parents signed informed consent before recruitment of their neonates in our study.

## 3. Results

In total, 134 neonates with perinatal hypoxia were enrolled in the study. One hundred and eighty-nine (189) healthy term neonates, formerly recruited, served as our control group [50]. Demographic and baseline characteristics of the research cohort and of the control group are presented in Table 1. None of the neonates of the hypoxic group fulfilled the criteria for birth asphyxia; all of them met the criteria of NRFS. Neonates with perinatal stress had a decreased birth weight, increased rate of meconium-stained amniotic fluid, lower 1-min. and 5-min. Apgar scores, and lower values of cord blood pH compared to healthy neonates. These differences were statistically significant. Moreover, statistical significance was also reported for delivery mode, since higher rates of instrumental delivery were observed in the hypoxic group.

Median values and IQR for NATEM parameters of newborns with perinatal hypoxia and healthy neonates are presented in Table 2. Comparison of NATEM values between the 2 groups of neonates revealed that hypoxic neonates had lower CT values (*p* < 0.05) and higher LI60 values compared to healthy neonates (*p* < 0.001).

After adjustment for confounding factors (birth weight, gestational age, gender, mode of delivery), we conducted multiple linear regression analyses and found that perinatal hypoxia was an independent risk factor for decreased fibrinolysis. On the contrary, perinatal stress did not have an impact on CT (Table 3).

The results of the Spearman correlation analysis of ROTEM values, with umbilical cord pH, lactic acid, and platelet counts, are depicted in Table 4. We observed a negative correlation between cord blood pH and LI60. Moreover, cord blood lactic acid was negatively correlated with CT and CFT and positively correlated with α-angle and LI60. Furthermore, a positive correlation between platelet count and A5, A10, MCF, and MCE was noticed, as well as a negative correlation between platelet number with CFT and LI60.

All neonates of the study population were observed for at least 3 days until discharge. None of them presented any clinically significant bleeding or thrombotic complication. Two cases of cephalohematoma following instrumental delivery and one case of stress ulcer were noted.

## 4. Discussion

In our study, the hemostatic status of neonates with perinatal hypoxia was investigated by applying NATEM assay to cord blood samples. Our results suggest that neonates with perinatal hypoxia have decreased fibrinolytic potential compared to healthy ones.

Perinatal hypoxia is marked by inadequate blood supply or oxygen delivery to or from the fetus during the intrapartum and peripartum period, which may result in hypoxia, hypercapnia, and acidosis. Hypoxic neonates are characterized by low Apgar scores, acidemia, and multi-organ dysfunction, while brain injury findings may also be noted [2]. Although our study group solely included neonates with perinatal stress, with none meeting the criteria for perinatal asphyxia, higher rates of meconium-stained liquor, lower Apgar scores in the 1st and 5th min., and lower umbilical cord pH values compared to those of the control group were noted. Additionally, in pregnancies affected by placental insufficiency, a compromised placenta restricts oxygen and nutrient supply to the fetus, thereby predisposing the infant to chronic perinatal hypoxia and growth restriction [57,58]. Correspondingly, although there were no statistically significant differences in gestational age between the 2 groups of our study, neonates with perinatal hypoxia had lower birth weight compared to controls.

The hemostatic and fibrinolytic systems are incompletely developed in newborns and gradually evolve through adulthood. In spite of this immaturity, the hemostatic system of healthy neonates remains functionally balanced, without thrombotic or hemorrhagic tendency [59,60,61,62,63]. However, in critically ill neonates, such as asphyxiated neonates, this functional equilibrium may be disturbed, making them prone to thrombotic and/or bleeding events [64]. In our study, the comparison between NATEM values in cord blood samples of neonates with perinatal hypoxia versus cord blood samples of healthy neonates revealed that, after adjusting for confounding factors, neonates with perinatal stress had decreased fibrinolytic potential, as expressed by higher LI60 values, compared to healthy ones. Changes in the balance of anticoagulant and fibrinolytic mediators in asphyxiated neonates resulting from lower natural inhibitors of coagulation levels and suppression of the fibrinolytic process may contribute to an exaggerated activation of the hemostatic mechanism, along with reduced fibrin lysis in the blood vessel [65,66,67]. In favor of this idea, animal and adult studies, or studies with human cell cultures, proposed several pathophysiologic mechanisms occurring at the tissue and cellular levels to attempt to explain the increased fibrin accrual and decreased fibrin dissolution in hypoxic conditions. Specifically, oxygen deprivation leads not only to the augmented release of tissue factor from circulating monocytes and endothelial cells, but also to the amplified expression of plasminogen activator inhibitor-1 (PAI-1), the most significant inhibitor of fibrin lysis, and to the suppression of plasminogen activator (PA) expression. These actions are largely mediated by transcription factors, such as early growth response-1 (Egr-1) and hypoxia-inducible factor 1-alpha (HIF-1a), which bind to the promoter region of relevant genes and stimulate their expression in low-oxygen conditions, disrupting the dynamic equilibrium between thrombus formation and clot lysis and tipping the balance in favor of enhanced net thrombotic activity [68,69]. Additionally, interleukin-1 (IL-1) is a potent inflammatory cytokine whose levels have been elevated in cases of perinatal hypoxia [70,71]. According to the literature data, IL-1 has been associated with increased PAI-1 production from the endothelial cells, further contributing to fibrinolysis shut-down [72,73]. Besides, thrombin activatable fibrinolysis inhibitor (TAFI), directly activated by thrombin to its active form, is another potent attenuator of fibrinolysis. Gursoy et al. studied TAFI levels in neonates born with meconium-stained and clear liquor. They reported that neonates from the meconium-stained liquor group had increased levels of TAFI and that there was also a negative correlation between blood pH and BE and TAFI levels [74]. Another study by Gursoy et al., concerning TAFI levels in neonates with respiratory distress syndrome (RDS), also came to the same conclusion; hypoxic neonates due to RDS had increased TAFI levels compared to the control group [75]. They hence concluded that increased TAFI levels may be due to acidosis and hypoxia, proposing an alternative path leading to depressed fibrinolysis in hypoxic neonates.

Data in the literature regarding the hemostatic status of hypoxic neonates vary, and this variability mainly arises from the differentiation of hemostasis tests used, the discrepancy in the population included, and the timing of patient enrollment on each occasion. Suzuki et al. [10] and Hathaway et al. [56] applied TEG in the umbilical cord blood of neonates with perinatal stress. They both found that R (reaction time) and K (kinetics time) showed no difference between neonates with perinatal hypoxia and healthy neonates, which is in accordance with our results. These two studies did not provide any data regarding LY30 (lysis at 30 min.) or LY60 (lysis at 60 min.). On the contrary, Golub et al. [55], who investigated the hemostatic status of neonates with birth asphyxia by performing TEG in neonatal blood samples during the first hour of life, noted that asphyxiated neonates had longer R and K and lower α-angle and MA (maximum amplitude) compared to healthy neonates. LY30 did not differ between the 2 groups. Konstantinidi et al. [53] performed EXTEM assay in neonatal blood samples of hypoxic and healthy newborns during the 2nd and 3rd day of life and described that newborns with perinatal hypoxia had a hypocoagulable profile and increased fibrinolytic activity, as expressed by longer CT and CFT and decreased A10, α-angle, MCF, and LI60 compared to healthy neonates. The divergence observed between our findings and the findings of Golub et al. and Konstantinidi et al. could be attributed to the fact that they used neonatal blood samples taken a few hours after delivery, instead of cord blood samples. Additionally, their study population included severely asphyxiated neonates, whereas only hypoxic neonates who did not fulfill the criteria for birth asphyxia were included in our research. We could therefore suggest that our results reflect the beginning of a cascade of events triggered by scarcity of oxygen that act cooperatively and disrupt the hemostatic equilibrium. If the hypoxic insult is of sufficient severity, or if the delivery is not completed quickly, an exaggerated activation of the hemostatic mechanism may ensue, predisposing neonates to consumption coagulopathy and disseminated intravascular coagulation (DIC), as is reflected by prolonged clotting times and increased fibrinolysis in TEG/ROTEM assays performed in neonatal samples [10,12]. Besides, studies with the standard coagulation tests in neonatal blood samples of severely asphyxiated neonates confirmed the higher rates of consumption coagulopathy in this subpopulation. Elsadek et al. [16] and Choudhary et al. [15] described prolonged PT, aPTT, and INR in asphyxiated neonates compared to healthy neonates. Additionally, other studies have reported prolonged clotting times, along with increased D-dimers and lower fibrinogen implying higher rates, of DIC in asphyxiated neonates [13,15,76]. Moreover, El Beshlawy et al. [77] and Canbal et al. [11] noted reduced levels of protein C, protein S, and antithrombin III in asphyxiated compared to healthy neonates, along with prolonged coagulation times and decreased fibrinogen levels, implying increased rates of DIC in the asphyxiated group as well.

Performing Spearman correlation analysis in our hypoxic group, umbilical cord pH was negatively correlated with LI60, and lactic acid values were negatively correlated with CT and CFT and positively correlated with α-angle and LI60. These results suggest an interrelationship between the severity of hypoxia, as reflected by the degree of metabolic acidosis, and NATEM variables, with hypoxia initially shifting the hemostatic balance towards hypercoagulation and decreased fibrinolysis. As outlined above, if hypoxia is prolonged and delivery is not performed promptly, before the onset of relevant clinical signs, the exaggerated initiation of the coagulation cascade and the interruption of fibrinolysis will eventually lead to consumption coagulopathy and probably DIC, and these hematological alterations seem to be influenced by the degree of the hypoxic insult. Data from the literature support our findings. Chadd et al. published that clotting times and FDPs (fibrin degradation products) were correlated to Apgar scores, and this association was statistically significant [13]. Similarly, Suzuki et al. found a statistically significant association between cord blood pH and FDPs [10]. Konstantinidi et al. compared asphyxiated and hypoxic neonates and found that asphyxiated neonates had a more intense degree of hemostatic alterations compared to hypoxic neonates [53]. Regarding fibrinolysis, in accordance with our findings, Gursoy et al. noted a negative correlation between cord blood pH and TAFI levels, indicating that fibrinolysis could be influenced by the degree of hypoxia as well [74]. Spearman correlation analysis also revealed a positive correlation between platelet count and A5, A10, MCF, and MCE and a negative correlation with CFT and LI60. Clot firmness is directly dependent on platelet count, fibrinogen concentration, and fibrin polymerization; therefore, a positive correlation between platelet number and A5, A10, MCF, and MCE could be anticipated [46]. Moreover, CFT largely depends on thrombin formation, along with platelet number and function, fibrinogen levels, and fibrin polymerization; hence the correlation found in our study between platelet count and CFT is thoroughly justified [45]. Regarding the correlation between platelet number and clot lysis, data in the literature are still conflicting, since platelets contain a variety of pro- and anti-fibrinolytic agents that could up- or down-regulate fibrinolysis [78,79].

According to the literature data, although numerous studies reveal derangement of hemostatic parameters in hypoxic neonates, the association between this laboratory evidence of hemostasis dysfunction and clinical thrombotic or hemorrhagic complications has not been established yet [80,81]. A considerable variability exists among institutions regarding the monitoring protocols of the coagulation status of hypoxic neonates. This uncertainty mainly arises from the dilemma of whether blood product transfusion therapy should aim at normalizing the conventional coagulation tests, or if it is preferable to adopt a more conservative approach by providing transfusion therapy only after bleeding events are observed [82,83]. Prolongation of clotting times, expressed by conventional coagulation tests, is a common finding in hypoxic neonates; however, these tests cannot provide information regarding the interaction of clotting factors with cellular participants in hemostasis or details concerning fibrinolysis; hence, their diagnostic capacity in predicting or diagnosing hemorrhage is poor. In contrast, ROTEM is a functional test that assesses the dynamics of clot formation and degradation in whole blood samples, and as a result, it seems to reflect accurately the in vivo hemostatic mechanism, taking into account the complex interplay between plasma proteins and cellular components [84]. NATEM assay, in particular, does not necessitate any reagent for activation of coagulation, it only requires calcium in order to reverse the effects of citrate in the collecting tube [45,49]. It is, therefore, a highly sensitive test in any endogenous element initiating the hemostatic mechanism, and may provide more precise insight into the in vivo coagulation cascade than EXTEM or INTEM. Besides, the use of a single reagent renders NATEM a more economical assay compared to different ROTEM assays. Although not firmly established, bibliographic data suggest that NATEM may be more effective in detecting fibrinolysis derangement in critically ill patients compared to EXTEM/INTEM [48,52]. Preliminary studies using viscoelastic tests for neonatal hemostasis assessment suggest their potential superiority over standard coagulation tests in predicting the true bleeding risk and guiding rational transfusion therapy, leading to a decrease in neonatal mortality and morbidity [21,85]. Therefore, NATEM could constitute a practical tool in the prompt identification of hemostasis derangement in hypoxic neonates prior to the onset of relevant clinical signs.

The present study has certain limitations. Our initial intention was to investigate the hemostatic profile of both asphyxiated neonates and neonates with NRFS; however, none of the neonates fulfilled the ACOG and AAP criteria for perinatal asphyxia, and all our study participants met the criteria for NRFS. Consequently, this may have restricted our ability to obtain statistically significant results where statistical trends were noticed and to associate our findings with possible clinical complications. However, neonates with NRFS constitute a distinctive subpopulation. They frequently manifest subtle and broad clinical signs that may mislead medical practitioners, leading to erroneous diagnoses and unnecessary interventions such as unwarranted laboratory investigations or indiscriminate antibiotic usage; they hence deserve the special attention of neonatologists. Additionally, we were not able to correlate our results with standard coagulation tests, because delayed cord clamping is a standard practice in our institution. Therefore, only a small volume of blood could be extracted in each case. Finally, our results warrant careful interpretation, since cord blood samples were used instead of neonatal blood samples, as minimal handling policy is followed in our institution. Nonetheless, based on bibliographic data, umbilical cord samples are equivalent to neonatal samples and could be used interchangeably on special occasions [86].

## 5. Conclusions

To the best of our knowledge, this is the first study assessing the hemostatic status of hypoxic neonates performing the NATEM method in cord blood. Hypoxic neonates seem to have a lower fibrinolytic potential relative to healthy neonates, as reflected by higher LI60 values. Our results indicate that NATEM may serve as a valuable tool for clinicians by enabling the early detection of fibrinolysis disorders, even prior to the manifestation of clinical symptoms, thus facilitating prompt diagnosis and timely treatment. Additional research is required to confirm our findings and to clarify the role of NATEM in the early diagnosis of hemostasis derangement, in the hemostasis monitoring protocols, and in the tailored treatment of this neonatal subpopulation.

## Figures and Tables

**Table 1:** Demographic characteristics of hypoxic and healthy neonates.

Characteristics	Hypoxic Neonates (N = 134)	Healthy Neonates (N = 189)		
	Median (Q1–Q3) or n (%)	Median (Q1–Q3) or n (%)	*p*-Value	OR (95% CI)
Birth weight (grams)	3150 (2890–3460)	3330 (3140–3530)	<0.0001	NA
Gestational age (weeks)	39 (38–40)	39 (39–40)	0.90	NA
Gender	Female (66/49.25%) Male (68/50.75%)	Female (86/45.5%) Male (103/54.5%)	0.57	0.86 (0.55–1.34)
Delivery mode	ND (40/29.85%) CS (64/47.76%) ID (30/22.39%)	ND (61/32.28%) CS (116/61.38%) ID (12/6.35%)	0.0001	NA
Maternal coagulation disorders	Yes (3/2.24%) No (131/97.76%)	Yes (11/5.82%) No (178/94.18%)	0.17	2.7 (0.74–9.87)
Amniotic fluid	Clear (102/76.12%) Meconium-stained (32/23.88%)	Clear (182/96.3%) Meconium-stained (7/3.7%)	<0.0001	0.12 (0.05–0.29)
APGAR (1st min.)	9 (8–9)	9 (9–9)	<0.0001	NA
APGAR (5th min.)	10 (10–10)	10 (10–10)	<0.0001	NA
Umbilical cord pH	7.3 (7.2–7.3)	7.4 (7.3–7.4)	<0.0001	NA

Abbreviations: CS, cesarean section; ID, instrumental delivery; NA, not applicable; ND, normal delivery.

**Table 2:** Comparison of NATEM values between hypoxic and healthy neonates.

	Hypoxic Neonates (N = 134)	Healthy Neonates (N = 189)	*p*-Value
CT	306 (235–360)	322 (250–391)	<0.05
CFT	94 (80–117)	97 (80–127)	0.36
A5	40 (36–44)	41 (36–45.5)	0.51
A10	50 (46–54)	51 (47–55)	0.32
MCF	57 (54–61)	58 (54–61)	0.76
α-angle	71 (67–74)	71 (65–74)	0.27
LI60	94 (92–96)	93 (91–95)	<0.0001
MCE	135 (118–159)	136.5 (118–158.5)	0.74

Abbreviations: CT, clotting time; CFT, clot formation time; A5, clot amplitude at 5 min.; A10, clot amplitude at 10 min.; MCF, maximum clot firmness; LI60, lysis index at 60 min.; MCE, maximum clot elasticity.

**Table 3:** Multiple linear regression analysis for NATEM values as dependent variables with hypoxia (adjusted for birth weight, gestational age, gender, and delivery mode).

	Beta (Coefficient)	Standard Error	*p*-Value
CT	−19.2	10.2	0.06
CFT	5	5.9	0.4
A5	−0.90	0.82	0.27
A10	−1	0.80	0.21
MCF	−0.35	0.72	0.63
α-angle	0.03	0.71	0.97
LI60	1.4	0.34	<.0001
MCE	−0.91	3.6	0.53

Abbreviations: CT, clotting time; CFT, clot formation time; A5, clot amplitude at 5 min.; A10, clot amplitude at 10 min.; MCF, maximum clot firmness; LI60, lysis index at 60 min.; MCE, maximum clot elasticity.

**Table 4:** Spearman correlation of NATEM values with laboratory results of hypoxic neonates.

	pH (UC)	Lac (UC)	PLTs (UC)
CT	0.01 0.8517	−0.29 0.0004	−0.02 0.888
A5	0.03 0.6489	0.12 0.1493	0.43 0.0003
A10	0.03 0.6486	0.10 0.2381	0.46 0.0001
CFT	0.02 0.6756	−0.18 0.0344	−0.31 0.0107
MCF	−0.03 0.5638	0.15 0.0745	0.43 0.0004
Alpha	−0.03 0.5798	0.21 0.0136	0.30 0.0158
LI60	−0.21 0.0002	0.18 0.0368	−0.28 0.0242
MCE	−0.03 0.537	0.15 0.0743	0.43 0.0003

Abbreviations: CT, clotting time; CFT, clot formation time; A5, clot amplitude at 5 min.; A10, clot amplitude at 10 min.; MCF, maximum clot firmness; Lac, lactic acid; LI60, lysis index at 60 min.; MCE, maximum clot elasticity; PLTs, platelets; UC, umbilical cord.

## Data Availability

The original contributions presented in the study are included in the article, further inquiries can be directed to the corresponding author/s.
